# Pivoting in the pandemic: how HIV-focused community-health workers addressed COVID-19 disparities

**DOI:** 10.3389/frhs.2026.1813009

**Published:** 2026-09-17

**Authors:** Sonjia Kenya, BreAnne Young, Olveen Carrasquillo

**Affiliations:** 1 Department of General Medicine, University of Miami Miller School of Medicine, Miami, United States; 2 Department of Public Health Sciences, University of Miami Miller School of Medicine, Miami, United States

**Keywords:** CHW model, community health workers, COVID-19, HIV, interventions, SoFlo CEAL, vaccination

## Abstract

**Introduction:**

The COVID-19 pandemic significantly overburdened the U.S. healthcare system and magnified pre-existing care delivery gaps in communities of color. In Miami-Dade County, Black residents account for 64% of AIDS-related deaths and experienced a COVID-19 mortality rate of 6.1%, compared to 3.9% among White residents. Community Health Worker (CHW) interventions represent a promising strategy to overcome social and economic barriers to care.

**Methods:**

Leveraging a decade-long partnership with nine community-based organizations, an established CHW network originally designed for HIV outreach, was adapted to address COVID-19 disparities in high-risk minority communities. From June 2021 through May 2023, this CHW coalition incorporated COVID-19 education and vaccination support into existing outreach protocols to ensure the approach remained aligned with community values.

**Results:**

During the 2-year intervention, the CHW network provided COVID-19 education regarding risk factors and vaccine safety to over 400,000 local residents. Furthermore, 8,672 individuals were screened for COVID-19, and 5,341 people were successfully referred for vaccination through these community-led efforts.

**Discussion:**

Findings support engaging CHWs to deliver interventions as an adaptable strategy to improve access to care and increase uptake of resources shared about COVID-19 screening and vaccination. By being respected and well-known by their neighbors, those who work in CHW-led interventions are uniquely positioned to promote awareness and dispel myths in highly vulnerable communities. This model demonstrates the value of utilizing trusted community peers trained in healthcare navigation to bridge gaps during public health emergencies.

## Introduction

The burden of morbidity and mortality posed by the COVID-19 pandemic was not shared equally (1, 2). Evidence suggests that disparities in COVID-19 outcomes were heavily rooted in socioeconomic inequities, many of which overlapped as drivers of HIV disparities among Black communities across the Nation (1, 2). As seen across other health conditions from diabetes to HIV, historically marginalized communities suffered the greatest impact. At the onset of the pandemic, Black patients in the U.S. were hospitalized at three times the rate of White patients, with death rates over twice as high (1, 2). In a matter of months, the stark disparities in COVID-19 morbidity and mortality rates that emerged in communities of color evoked a pattern of poor outcomes parallel to that of HIV in minority populations.

Miami Dade County, in particular, has experienced some of the greatest racial disparities in HIV outcomes (3). In 2024, Black people comprised just 17% of County residents yet accounted for nearly half of people living with HIV and 64% of AIDS-related deaths, indicating critical gaps in the HIV care continuum among this population (3). Similarly, as of April 2020, Black residents in Miami accounted for 21% of COVID-19 related deaths, resulting in a mortality rate of 6.1% compared to 3.9% among White people (2, 3). The parallels between these health disparities highlighted persistent injustices but also offered an opportunity to adapt efficacious HIV-focused initiatives for this population, to also address COVID-19.

One promising strategy to overcome social and economic barriers to care has been the use of Community Health Worker (CHW) interventions (4–6). Identified by the Department of Homeland Security as “essential infrastructure workers” during the response to the COVID-19 emergency, CHW-led interventions played a critical role in both public health and safety as well as community well-being (7). By hiring people from communities of focus who are respected and well-known by their neighbors, a deep understanding of the health and social needs of the community are embedded in CHW initiatives and make them effective at improving local health outcomes.

Throughout the pandemic, CHW models transitioned from showing theoretical promise to serving as a documented, frontline pillar of health equity (8). Comprehensive systematic reviews demonstrate that pre-existing CHW infrastructure, particularly frameworks originally designed for tracking and managing infectious diseases like HIV, were rapidly and successfully pivoted to deploy responsive pandemic surveillance, contact tracing, and education (8, 9). In communities of color disproportionately impacted by medical mistrust, systemic exclusion, and targeted misinformation, those employed in CHW-led interventions acted as essential insider messengers (8–10).

Empirical studies show that targeted CHW-led education and community-driven toolkits significantly mitigated vaccine hesitancy, accelerated health literacy, and fostered increases in both COVID-19 testing and vaccine uptake among highly vulnerable and historically marginalized populations (10, 11). Consequently, leveraging the trusted infrastructure of established CHW networks offers a rigorous, validated pathway for adapting existing HIV-focused health equity frameworks to counter unanticipated health crises, such as the COVID-19 pandemic.

As previously described, CHW-led interventions led by this team have established a robust network of community partners to improve access to health education, HIV testing, care linkage, and medications among Black adults in Miami's high-risk neighborhoods (12–15). When the pandemic emerged, this existing CHW network was charged with addressing COVID-19 disparities using a similar model to serve this same Black communities plagued by the County's greatest HIV disparities. To support this work, UM received a grant in June 2021 to provide community-based COVID-19 awareness and vaccination opportunities in South Florida's underserved minority communities with the lowest COVID-19 vaccination rates. Adapted from our CHW model, the South Florida Community Engaged Alliance Against Covid-19 Disparities (SoFlo CEAL) aimed to increase COVID screening, reduce vaccine hesitancy, and promote vaccine uptake among high-risk minority populations with limited access to the formal healthcare system. This manuscript describes CHW-led interventions that rapidly pivoted during the pandemic from addressing HIV disparities to focusing on COVID-19 outcomes.

## Materials and methods

This work was deemed exempt/not human subject research by the University of Miami Institutional review Board. The implementation of the COVID-19 pivot occurred across two distinct phases, transitioning from a broad mobilization involving nine partners in Year 1 to a consolidated model in Year 2 with just three partners. The project was funded for both years; however, different levels of funding were provided for each year.

Between June 1, 2021, through May 31, 2022, a total of nine organizations, in addition to UM, were funded. As COVID-19 cases fell dramatically, funding continued for UM, who oversaw the efforts of three partner organizations, through May 31, 2023.

Between June and July 2021, the UM research team established binding agreements with nine HIV community service organizations that managed existing CHW programs (Figure 1). These agreements focused specifically on provision of services related to COVID-19. Each organization served a resource-poor geographic region or distinct vulnerable community (i.e., historically Black neighborhoods, undocumented migrant workers, etc.) in South Florida. Partner organizations utilized CHW models to increase access to social support and clinical resources in their communities of focus, with a major focus on addressing HIV disparities.

**Figure 1:**
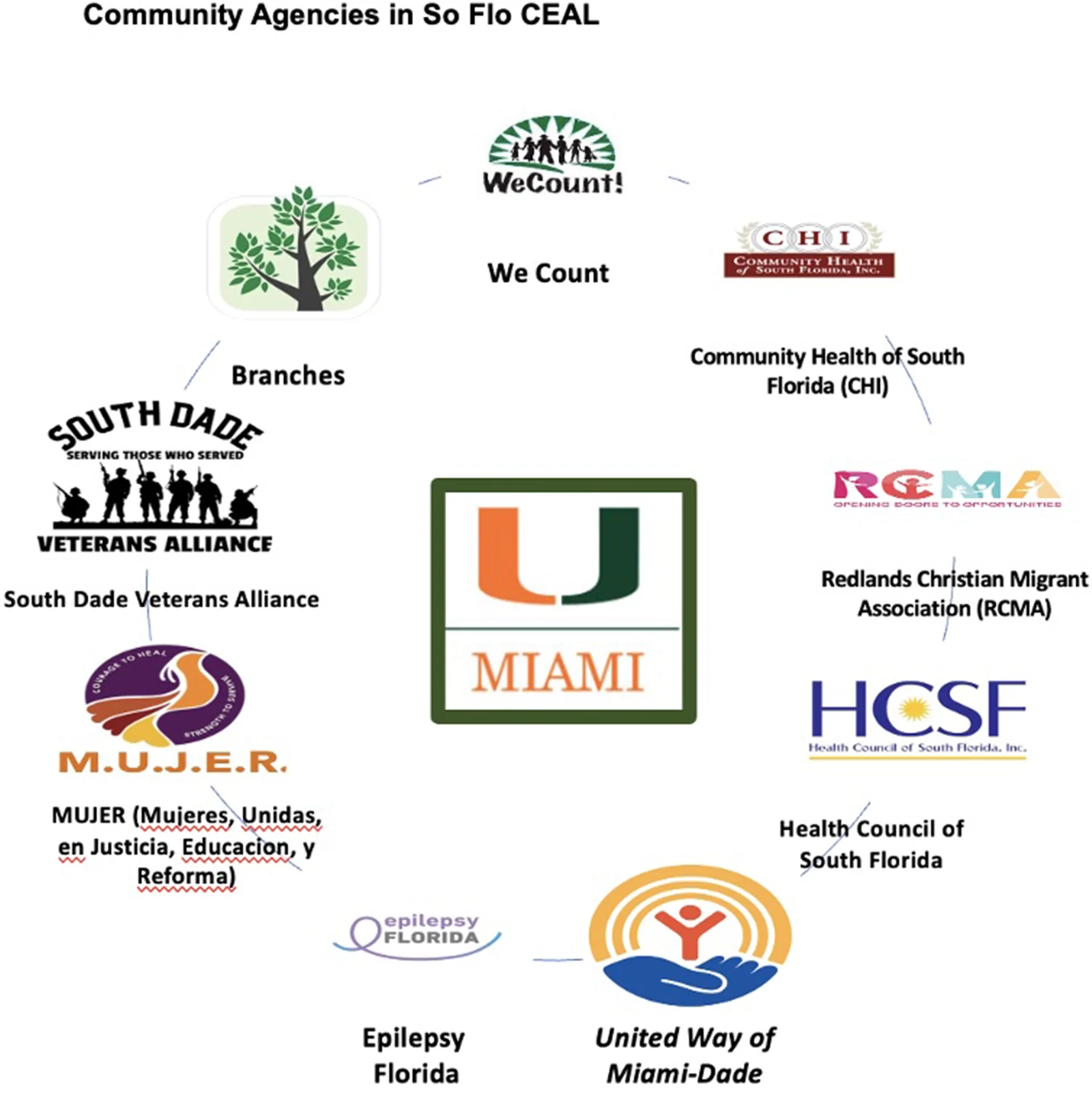
Community agencies in SoFlo CEAL.

Overall project oversight was conducted by UM researchers who led trainings for each organization, provided documentation templates to record project activities, and facilitated a monthly meeting with all community partners to report outcomes, address challenges, and develop communication strategies to prevent duplication of efforts among organizations and create collaborative synergy.

To rapidly equip the coalition to address COVID-19 disparities in highly impacted neighborhoods, the University of Miami Infectious Disease Research Team developed a tailored, culturally responsive COVID-19 training curriculum for CHW initiatives. This curriculum was specifically designed to leverage the existing community trust held by HIV-focused CHW-led interventions and pivot their expertise toward pandemic mitigation. The training framework integrated local public health policies regarding personal space and social distancing, the most up-to-date federal and local recommendations for COVID-19 screening and vaccinations, and evidence-based communication strategies for engaging vaccine-hesitant individuals regarding the benefits of immunization. The goal of the training was to prepare laypeople working as CHWs to educate community members about COVID-19 safety, screen community members for COVID-19, and increase uptake of COVID-19 vaccinations.

The University of Miami research team initially conducted this standardized training via zoom with all of the leadership and training coordinators from the nine participating community agencies. Following this initial phase, the research team provided each agency with a complete, adaptable training module. This enabled a sustainable train-the-trainer model, allowing each individual agency to internally train their respective teams of CHWs. Equipped with these standardized competencies, CHW initiatives were deployed to educate community members on COVID-19 health literacy, conduct frontline screenings, and actively facilitate vaccination referrals to increase vaccine uptake among vulnerable populations.

Each community agency trained and managed their existing CHW intervention team. Project activities varied by agency, were offered in three different languages (Spanish, Haitian Creole, and English), and tailored to the communities being served. Activities included physical community workshops, popup screening and vaccination events, participation in community health fairs, calls/texts with community members and social media engagement.

Aligned with the project aims, data was collected to measure the: (1) number of people engaged through various types of outreach (face to face, virtual phone calls, text messages, social media); (2) number of people screened for COVID-19, and (3) number of people who received personalized assistance to an accessible vaccination site and making an appointment, when available. Data was provided to UM researchers by community agencies as de-identified operational metrics (e.g., total counts of individuals reached, screened, and referred) and the analyses were limited to aggregating these anonymized metrics.

The amount of time CHW teams spent performing specific tasks was assessed qualitatively. On a monthly basis, CHW administrators at each agency asked their staff to indicate the amount of time they spent performing specific tasks. This occurred during routine meetings when supervisors met with CHW teams to review their workload, as these were additional duties they were asked to perform as part of their full-time position. Specifically, UM researchers tasked CHW supervisors with recording the amount of time each CHW spent on specific activities and calculating the total amount of effort their entire CHW staff spent on each outreach category. This data was reported during the monthly meetings among all agencies and the UM research team. Monthly meetings were also used to coordinate collaborative community-based activities involving multiple agencies and address challenges. For example, when one agency reported barriers providing a specific service, such as facilitating vaccinations, other agencies and the UM research team collectively brainstormed potential strategies to achieve project aims.

While each agency conducted various activities, all CHW-led interventions were specifically focused on education, screening, and vaccination referrals. Based on each organization's specific areas of expertise, partners that provided different services were encouraged by the UM research team to coordinate complementary interventions whenever possible. For example, when one agency conducted an education-focused workshop, a partner agency would be on-site to facilitate screenings and vaccinations, or referral to vaccinations. Similarly, when a CHW team led a vaccination pop-up event, a CHW team from a different agency would provide education about accessing the latest COVID-19 treatments and guidance to reduce transmission.

At community events, CHW teams engaged with individuals who attended such events, including some of their prior clients who they informed about accessible COVID-19 support. Those who worked as CHWs were routinely contacted by phone/text by people they previously helped address other social or health issues. In these circumstances, CHW teams were trained to answer specific questions directly, provide text links to basic COVID-19 health information, offer personalized education or screening care, educate individuals about community-based COVID-19 education, screening, and vaccination events, and when needed, refer individuals to specific care sites.

In addition to in-person events, the UM research team led a robust online outreach campaign that involved daily social media posts on Instagram and Facebook focused on increasing COVID-19 knowledge, encouraging vaccination uptake, and highlighting the in-person COVID-19 screening and vaccination events hosted by community partners. Each community agency was also encouraged to post COVID-19 information and promote their activities related to the SoFlo CEAL project. All partners were asked to tag relevant posts with #SoFloCEAL. Metrics provided by each social media platform indicated the number of people reached and engaged on each post.

As stated above, each community agency used documentation templates provided by the UM research team to report outcomes monthly. Data collected included total counts of individuals reached, screened, and provided with vaccination assistance. Individuals reached was defined as the number of people CHW programs exchanged COVID-19 information, and provision of vaccination assistance, including providing operating hours, and/or making appointments for vaccination when available. At times, screening occurred at pop up COVID-focused outreach events, where onsite vaccination was available. At these events, vaccination assistance also included accompanying individuals to receive a vaccine from an onsite provider.

Data was analyzed to quantify in-person, digitally mediated, and telecommunication activities:

- In-person was defined as direct physical outreach and included outreach that occurred through physical, community-anchored efforts (such as community health fairs, pop-up screenings, and public vaccination events). Traditional written COVID-19 educational pamphlets were also included in this category.
- Digitally mediated was defined as remote or virtual channels, including social media platforms.
- Telecommunication activities were defined as direct telephone calls, text messaging, and video consultations.
- Uncategorized/multiple contact points represented a very small number of encounters. They were defined as provision of information and support via multiple communication pathways such as in-person and digitally mediated. As CHWs involved in this program had a history of providing HIV support to the population of focus, there were times when they provided COVID-19 focused information in addition to HIV-focused support.

## Results

Year 1 (June 2021—May 2022): This rapid scale-up phase engaged nine community partners to train 121 adult CHWs. These teams conducted 1,319 community events, both direct physical outreach and digitally mediated outreach. Year 2 (June 2022—May 2023): The program transitioned to a consolidated model featuring three core community partners conducting 358 targeted events.

Analysis of the programmatic outreach channels (Figure 2) reveals a highly adaptive, multi-modal communication framework, that included in-person, digitally mediated, and telecommunications strategies. Rather than relying solely on traditional outreach, the CHW teams successfully leveraged a hybrid model of digital and physical engagement:

- Direct Physical Outreach (58%): 29% of physical outreach occurred at community efforts and 22% through individualized, in-person contact. Traditional written COVID-19 educational pamphlets represented the smallest portion of outreach at 7%.
- Digitally Mediated Outreach (28%): Nearly 30% of digitally mediated outreach occurred through social media engagement via the #SoFloCEAL campaign. In this category, direct messages from community accounted for 22% of outreach.
- Telephonic & Video Support (11%): This outreach included direct telephone calls, text messaging, and video consultations.
- Uncategorized/Multiple Contact Points (3%): This category represented those who received information and support via multiple communication pathways such as in-person and digitally mediated.

**Figure 2:**
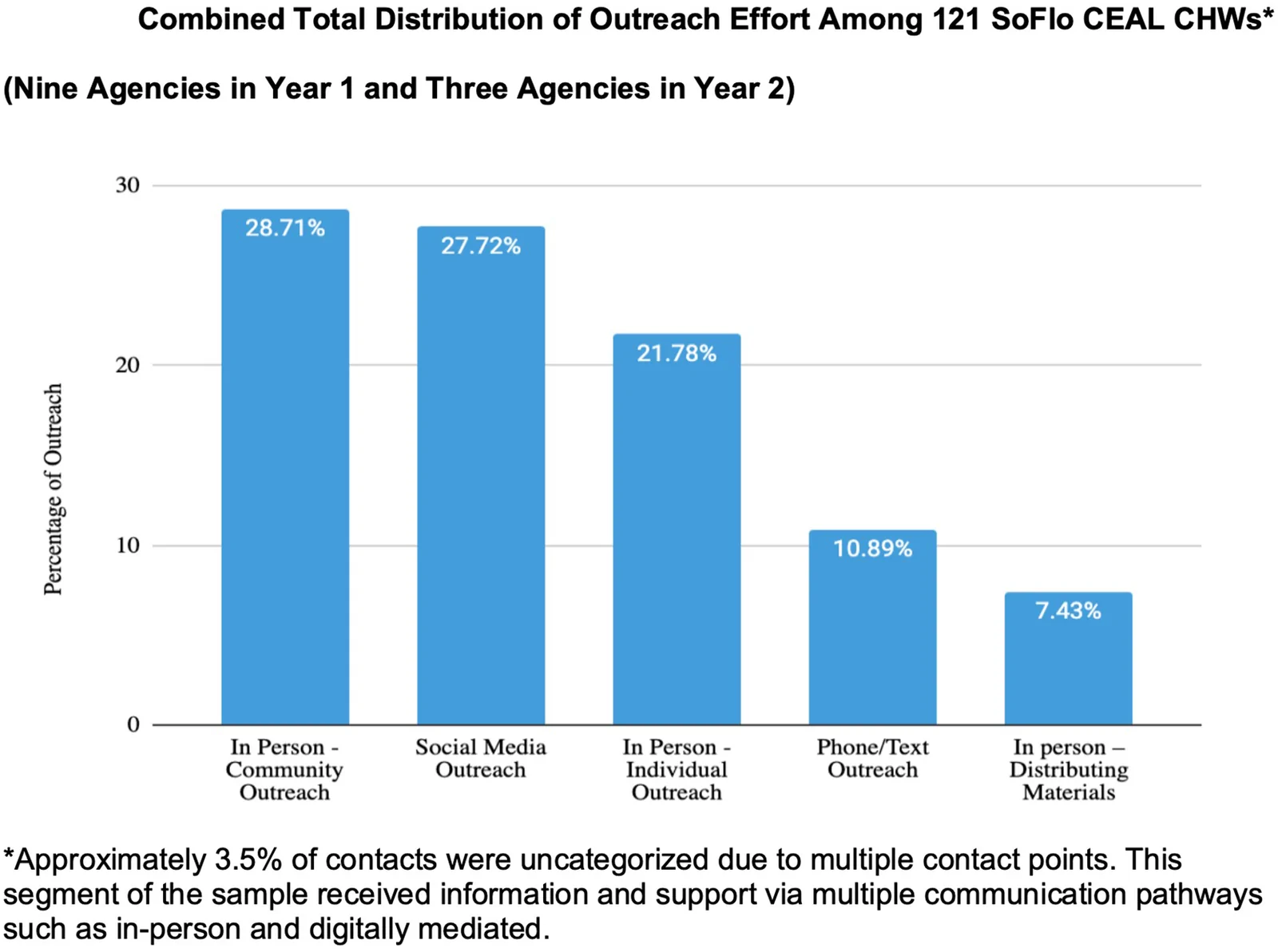
Distribution of outreach effort among 121 CHWs across nine agencies.

As shown in Table 1, Year 1 focused heavily on mass information campaign distribution, reaching approximately 400,000 individuals split equally between in-person and online platforms. This phase successfully referred 2,845 individuals to screenings and educated 3,643 individuals about resources to obtain COVID-19 vaccination. In Year 2, despite a 70% reduction in partner organizations and an expected drop in raw informational reach, the program demonstrated highly optimized referral pathways. Most notably, those who obtained COVID-19 screening more than doubled to 5,827 individuals (Table 1). This upward shift, a 105% increase in screening with only three partners, coincided with major public health advancements in COVID-19 screening and vaccination processes, as well as public messaging to combat vaccination hesitancy. Such improvements likely contributed to outcomes resulting from the work described herein and highlights how ta consolidated CHW network can successfully evolve to align with advancements within the public health infrastructure.

**Table 1:** Data overview of engagement.

Assessed intervention metrics	Year 1	Year 2	Total
Staff (CHWs) trained	121	N/A	121
Number of events	1,319	358	1,677
Outreach hours	298	N/A	298
Physical outreach			232,381
Individuals informed (community health fairs, pop-up screenings, public vaccination events, and education pamphlets)	197,433	21,115	218,548
Individuals given resources about vaccination events	3,643	1,698	5,161
Individuals screened	2,845	5,827	8,672
Digital outreach	205,617	28,241	233,858
Use of #SoFloCEAL			65,480
Direct messages			14,406

As reflected in Table 2, this distribution underscores that the HIV-focused CHWs successfully translated their historical expertise in discrete, trust-based, and highly personalized communication into the digital sphere, utilizing direct messaging and social media as critical virtual venues for pandemic response.

**Table 2:** Comparative data table year 1 and year 2.

Metric	Year 1: 9 partners ( + UM)	Year 2: 3 partners ( + UM)	Relative % change
Direct physical outreach (community health fairs, pop-up screenings, public vaccination events, and education pamphlets)	197,433	21,115	−89%
Digitally mediated outreach (remote or virtual channels, social media platforms, direct phone calls, text messaging, video consultations)	205,617	28,241	−86%
Vaccination referrals	3,643	1,698	−53%
Individuals screened screened	2,845	5,827	+105%

## Discussion

The findings of this study demonstrate that the primary value of a CHW workforce during a crisis lies not just in their clinical reach, but in their ability to be flexible in redirecting their expertise to address other health conditions. The main contribution of this research is the empirical demonstration that an existing, specialized CHW infrastructure can be rapidly and effectively pivoted during a public health emergency to mitigate escalating health disparities. Rather than constructing emergency outreach frameworks from scratch, an approach often plagued by administrative delays and community resistance; leveraging an established network of trusted messengers allowed for a swift, proactive response to the COVID-19 pandemic.

The evolution of our CHW intervention from Year 1 to Year 2 illustrates a transition from a broad-scale emergency response to refined, efficacious strategies that fostered rapid provision of education, screening, and vaccination uptake. While Year 1 focused on mass information dissemination to combat widespread pandemic uncertainty, Year 2 achieved a remarkable 105% increase in individuals seeking COVID-19 screenings despite a 70% reduction in partnering organizations.

This shift from nine partners screening 2,845 individuals in Year 1 to three partners screening 5,827 individuals in Year 2 is characteristic of effective community health programs as they become more established in their geographic areas of focus. Crucially, this evolution mirrors the historical trajectory of HIV outreach, which transitioned from early, broad-based awareness campaigns to highly targeted, low-barrier screening and linkage-to-care models. The surge in Year 2 screenings occurred simultaneously with significant improvements in the public health testing infrastructure throughout the progression of the pandemic. Similar to how HIV screening has become more accessible since the virus emerged, increased COVID-19 screening in our sample suggests that HIV-focused CHWs can successfully translate their existing competencies in diagnostic-focused outreach to support improved diagnostic accessibility in the COVID-19 landscape. Furthermore, the high rate of digitally mediated outreach, particularly the 22% of encounters occurring via direct messages, demonstrates that the digital pivot from Year 1 to Year 2 was not merely a passive broadcasting strategy, but an active, hybrid avenue for maintaining personalized, trust-based clinical referrals during times of physical restriction.

It was particularly advantageous that the CHW teams involved were already trained to address HIV, an infectious disease for which specific knowledge and protections are required to prevent transmission. Their expertise working within resource-limited neighborhoods, serving as a liaison between community-members and life-saving healthcare, and facilitating rapid testing coupled with immediate education, screening, and, for those who screened positive, accompanying them to same day confirmatory testing, prepared the existing CHW programs to quickly pivot towards addressing COVID-19. Simultaneously, the highly infectious nature of COVID-19, compounded by the medical community's lack of experience with this specific infectious agent, also forced CHW team leaders to explore untested strategies, such as providing education to families about isolating specific household members actively infected with COVID-19.

As COVID-19 risk dramatically declined, and more efficacious treatments became available, project funding ended at the end of Year 2. Thereafter, most CHW teams involved in the project returned to providing HIV-focused care. The knowledge and experience they gained during the pandemic strengthened their capacity to provide holistic support to people living with HIV, as they were equipped to provide individualized support regarding COVID-19 screening, testing, and treatment.

While outcomes from this work cannot be generalized to the entirety of South Florida, the data reported herein shows the SoFlo CEAL initiative fostered increased COVID-19 testing from June 2021 to May 2023. By building on an active HIV outreach infrastructure, this project effectively bypassed the traditional start-up lag that characterizes crisis-driven public health initiatives.

This strategy is strongly supported by broader pandemic literature, which confirmed on a global scale that public health systems utilizing pre-existing CHW frameworks, particularly those originally optimized for infectious diseases like HIV and tuberculosis, achieved significantly faster deployment and higher community compliance than newly assembled emergency outreach teams (8–10, 16). The trust equity accumulated by these networks prior to the pandemic acted as an irreplaceable public health currency, neutralizing the historic medical mistrust that heavily hindered traditional institutional rollouts.

This structural maturity and refinement are explicitly highlighted by the increased screening outcomes in Year 2 of the initiative. Despite a significant funding-driven contraction from nine community partners down to three, the total number of individuals successfully linked to screening nearly doubled from 2,845 in Year 1 to 5,827 in Year 2. This paradox suggests that as the remaining CHW coalition networks became well established, their localized navigation strategies became more efficient. Furthermore, it reflects the adaptive nature of the workforce alongside the evolving trajectory of the pandemic: as vaccines became widely accessible, the CHW initiatives pivoted from broad, volume-driven awareness campaigns to highly granular, individualized navigation to address vaccine hesitancy and dispel evolving misinformation (8, 17). This pattern of optimized efficiency amidst resource constraints mirrors multi-country pandemic evaluations, which found that as CHW networks matured throughout the COVID-19 crisis, they shifted from simple clinical tasks to complex case management and structural navigation (17). This maturation allowed mature CHW networks to maximize their public health impact and achieve greater community saturation even when facing sudden funding contractions or systemic bottlenecks (16, 17).

Qualitative feedback from our organizational partners during the monthly check-ins underscores that establishing physical or technical access to healthcare resources is only half the battle. The persistent skepticism and acute distrust of formal healthcare systems reported by frontline CHWs are not isolated anomalies; rather, they represent the rational, historic consequences of systemic health inequities within marginalized communities. By operating directly within neighborhoods and outside traditional clinical walls, CHW initiatives effectively function as a buffer of trust. They addressed pervasive myths, frequently amplified by social media channels, in a highly tailored manner that traditional, top-down government public service announcements simply cannot replicate.

Ultimately, the parallels between HIV and COVID-19 disparities within the Miami-Dade region are profound. Both pathogens exploit identical systemic fissures: housing instability, transportation barriers, and the digital divide. By utilizing an established HIV infrastructure, this initiative successfully demonstrated that public health emergency preparedness should not be treated as a series of isolated, disease-specific interventions. Instead, true pandemic resilience requires continuous, sustained investment in the human infrastructure of community health workers, ensuring a permanent mechanism is always in place to be pivoted whenever the next crisis emerges.

This research was limited by resource constraints and the transient nature of grassroots outreach. Specifically, the SoFLo CEAL initiative lacked resources to train CHWs to facilitate on-the-spot COVID-19 testing and/or vaccinations. Further, as none of the community agencies had a built-in mechanism for long-term, individual follow-up, CHW interactions were often limited to a single point of contact with community members. While each agency tracked vaccine referrals and some partner organizations also administered vaccinations directly, the total number of vaccinations received could not be verified due to health information privacy protections and the lack of clinical follow-up infrastructure. Reports from each partner agency quantified vaccinations based on self-reported data from individuals that received care from CHW teams within the coalition.

## Conclusion

The SoFlo CEAL initiative demonstrated that the Community Health Worker model is more than a supplementary tool; it is an essential component of health equity. By successfully pivoting a network designed for HIV advocacy to address the COVID-19 pandemic, we observed that trusted messengers can effectively bridge the gap between marginalized populations and formal healthcare systems.

The challenge remains to sustain this infrastructure. Dismantling these networks once a crisis fades only guarantees that we will be starting from scratch when the next disparity-driven health threat emerges. For communities like those in Miami-Dade, where the burden of HIV remains a daily reality, the CHW model offers a blueprint for a more resilient, integrated, and empathetic public health future.

## Data Availability

The original contributions presented in the study are included in the article, further inquiries can be directed to the corresponding author.
